# Multi-gene signature of microcalcification and risk prediction among Taiwanese breast cancer

**DOI:** 10.1038/s41598-020-74982-1

**Published:** 2020-10-26

**Authors:** Hsin-Tien Tsai, Ching-Shui Huang, Chao-Chiang Tu, Chih-Yi Liu, Chi-Jung Huang, Yuan-Soon Ho, Shih-Hsin Tu, Ling-Ming Tseng, Chi-Cheng Huang

**Affiliations:** 1 Division of General Surgery, Department of Surgery, Cathay General Hospital, Taipei, Taiwan; 2 Department of Surgery, School of Medicine, College of Medicine, Taipei Medical University, Taipei, Taiwan; 3 Department of Surgery, Fu-Jen Catholic University Hospital, New Taipei, Taiwan; 4 School of Medicine, College of Medicine, Fu-Jen Catholic University, New Taipei, Taiwan; 5 Division of Pathology, Cathay General Hospital Sijhih, New Taipei, Taiwan; 6 Department of Medical Research, Cathay General Hospital, Taipei, Taiwan; 7 Department of Biochemistry, National Defense Medical Center, Taipei, Taiwan; 8 TMU Research Center of Cancer Translational Medicine, Taipei Medical University, Taipei, Taiwan; 9 Taipei Cancer Center, Taipei Medical University, Taipei, Taiwan; 10 Department of Medical Laboratory, Taipei Medical University Hospital, Taipei, Taiwan; 11 School of Medical Laboratory Science and Biotechnology, College of Medical Science and Technology, Taipei Medical University, Taipei, Taiwan; 12 Division of Breast Surgery, Department of Surgery, Taipei Medical University Hospital, Taipei, Taiwan; 13 Comprehensive Breast Health Center, Taipei Veterans General Hospital, No. 201, Sec. 2, Shipai Rd., Beitou District, Taipei City, 11217 Taiwan, ROC; 14 School of Medicine, College of Medicine, National Yang-Ming University, Taipei, Taiwan

**Keywords:** Cancer, Computational biology and bioinformatics, Genetics

## Abstract

Microcalcification is one of the most common radiological and pathological features of breast ductal carcinoma in situ (DCIS), and to a lesser extent, invasive ductal carcinoma. We evaluated messenger RNA (mRNA) transcriptional profiles associated with ectopic mammary mineralization. A total of 109 breast cancers were assayed with oligonucleotide microarrays. The associations of mRNA abundance with microcalcifications and relevant clinical features were evaluated. Microcalcifications were present in 86 (79%) patients by pathological examination, and 81 (94%) were with coexistent DCIS, while only 13 (57%) of 23 patients without microcalcification, the invasive diseases were accompanied with DCIS (χ^2^-test, *P* < 0.001). There were 69 genes with differential mRNA abundance between breast cancers with and without microcalcifications, and 11 were associated with high-grade (comedo) type DCIS. Enriched Gene Ontology categories included glycosaminoglycan and aminoglycan metabolic processes and protein ubiquitination, indicating an active secretory process. The intersection (18 genes) of microcalcificaion-associated and DCIS-associated genes provided the best predictive accuracy of 82% with Bayesian compound covariate predictor. Ten genes were further selected for prognostic index score construction, and five-year relapse free survival was 91% for low-risk and 83% for high-risk group (log-rank test, *P* = 0.10). Our study suggested that microcalcification is not only the earliest detectable radiological sign for mammography screening but the phenomenon itself may reflect the underling events during mammary carcinogenesis. Future studies to evaluate the prognostic significance of microcalcifications are warranted.

## Introduction

Major efforts have been made for the prevention and management of breast cancer in the last century. Risk factors of breast cancer are rarely modified; therefore, the task of breast cancer prevention should emphasize early detection and appropriate treatment. The results of early detection are summarized among population-based trials^1^; early detection of breast cancer by mammography was proved to lower breast cancer mortality by one-third. In Taiwan 41% mortality reduction was reported from a 1.5 million population-based study^2^.

Experiences learnt from screening studies have established mammography as the only valid imaging modality for breast cancer early detection and subsequent mortality reduction^3^. The detection ability of mammography primarily relies on the identification of microcalcification, which represents ectopic mammary mineralization. Other distinguishingly mammographic features telling malignant from benign conditions include speculated mass, focal asymmetry, architectural distortions, and lesions with rapid progressions^4^.

Histologically there are two kinds of microcalcifications; type I microcalcification is made up of calcium oxalate (CaC_2_O_4_·2H_2_O) while for type II, hydroxyapatite (Ca_10_(PO_4_)·6H_2_O) is the main composition^5,6^. In appearance, type I microcalcification is amber while type II is gray to white. Under haematoxylin and eosin (H&E) dyes, type II microcalcification appears purple, whereas type I is rarely stained. Inspected with light microscopy, type I microcalcification is partial transparent while type II is opaque. Illuminated with polarized light, type I microcalcification is birefringent, which is not true for type II^7^. The major clinical significance of type I and II differentiation comes from the observation that type I microcalcification is accompanied with predominately benign lesions while associated lesions are half benign and half malignant for type II microcalcification^8^.

The absolute number, morphology, and distributions of microcalcifications on mammography are major deterministic features to tell suspicious from benign lesions, and standardized algorithm has been purposed to enhance diagnostic accuracy and reduce inter-observer variability such as the Breast Imaging-Reporting and Data System (BI-RADS) acronym^4,9^. From literature reviews, candidate genes involving in microcalcification formation and genes with molecular aberrations have been reported, namely *SPARC*, *SPP1*, *IBSP*, *NFKB1*, *NFKB2*, *REL*, *RELA*, *RELB*, *FOS*, *MYC*, *IL1B*, *CXCR4*, *MMP-1*, *CTGF*, *FGF5*, and *IL11* (more details in “Methods” section, ref. 10–25).

Whether microcalcification is merely a radiographic phenomenon during rapid mammary evolution or is itself an active process contributing to breast carcinogenesis remains inconclusive^6,10^. The high-throughput microarrays are advocated to capture whole-transcriptome during a single hybridization, and breast cancer is one of the most extensively assayed human malignancies. Ironically, rarely has it been reported regarding messenger RNA (mRNA) abundance associated with microcalcification. In current study, we evaluated the transcriptional profiles associated with microcalcification for Taiwanese breast cancer with a multi-gene signature derived.

## Materials and methods

This study was reviewed and approved by Institute Review Board of Cathay General Hospital. All research was performed in accordance with relevant guidelines/regulations. Informed consent was obtained from all participants after explanation by investigators (CCH and CSH). Some breast cancer samples which had been reported previously were also enrolled in current study^11^. Microarray data in current study was deposited in Gene Expression Omnibus (GEO) with the access number GSE146558.

### Breast cancer samples

Breast cancer samples were prospectively collected during surgery. Cancerous breast tissues were snap-frozen and stored in liquid nitrogen at − 80 °C with RNAlater reagent (Qiagen, Germantown, MD) within 30 min after surgery. Frozen samples were cut into 1–2-mm thick slices, and slices with more than 90% of cancer content were selected for microarray experiments.

Pathological features were retrieved from chart reviews. Estrogen receptor (ER) and progesterone receptor (PR) positivity was defined as the presence of at least 10% of nuclei with positive immunohistochemical (IHC) stains. Patients with low ER and PR IHC stains (defined as 1–9% of positive nuclei staining) were not enrolled as patients with low hormone receptor status were excluded in previous study^11^. American Society of Cancer Research (ASCO) and College of American Pathologists (CAP) guidelines were followed for determining human epidermal growth factor receptor 2 (HER2) status: IHC 3+ and IHC 2+ with fluorescence in situ hybridization (FISH) amplification were considered as HER2 overexpression. The presence of microcalcification, accompanied ductal carcinoma in situ (DCIS), and comedo-necrosis was ascertained from pathological report. In brief, microcalcification was detected as magenta to purple granules from routine H&E-stained sections based on the morphology^12^. All pathological examinations were carried out by one qualified pathologist (CYL) and more than 90% of cancerous tissue was a prerequisite for downstream experiments.

### Microarray experiment

Total RNA was extracted from frozen specimens using TRIzol reagent (Invitrogen, Carlsbad, CA). RNA was purified using RNeasy mini kits (Qiagen, Valencia, CA) and RNA integration was checked by gel electrophoresis. The Affymetrix GeneChip Human Genome U133 plus 2.0 (Thermo Fisher Scientific, Santa Clara, CA) was used for microarray experiments. Hybridization and scanning were performed according to the standard Affymetrix protocol. Image scanning was performed using a GeneChip Scanner 3000 (Thermo Fisher Scientific, Santa Clara, CA) with scanned images processed by GeneChip Operating Software and Affymetrix’s Microarray Suite software to generate detection p values. The Robust Multichip Average (RMA) algorithm, which consisted three steps of background adjustment, quantile normalization, and final summarization, was applied for perfect match probe signals within the study^13^. For multiple probe sets corresponding to single gene, probe sets were reduced to one per HUGO (Human Genome Organisation) gene symbol by using the most variable probe set measured by inter-quadrant range across all arrays^14^.

### Relevant genes for microcalcification

Through extensive literature reviews, genes relevant to the formation, distinguishing features or molecular aberrations of microcalcification were identified. Using IHC staining, Scimeca et al. postulated that epithelial-mesenchymal transition (EMT) may take place during breast carcinogenesis, and the capacity of producing microcalcifications is acquired during such process^10^. They identified vimentin, bone morphogenic protein-2 (BMP-2), β2-microglobulin, β-catenin, and osteopontin (OPN) as tissue mineralization or mesenchymal phenotype markers investigated with IHC. Through microarray experiments, Bellahcène et al. demonstrated ectopic mRNA transcription of bone extracellular matrix proteins including bone sialoprotein (BSP), OPN, and osteonectin (OSN) in osteotropic breast cancers^15^. It deserves notice that BSP is supposed to initiate hydroxyapatite deposition and recruit osteoclasts before crystal resorption during the early phase of bone matrix mineralization^16^. In addition, BSP expression, when assayed by both immunoperoxidase and polyclonal antibodies, was correlated with bone metastases and poor survival^16–18^.

Downstream effectors of hydroxyapatite include protein kinase C, nuclear factor-κB, proto-oncogenes c-fos and c-myc, all of which are associated with subsequent mitogenesis and proliferation from in vitro cell culture system and with corresponding antibodies^19^. In addition, up-regulation of matrix metalloproteinases (MMP)-2, -9, and -13 by hydroxyapatite has been observed in mammary epithelial cell lines by Western blotting^20^. Over-expression of MMPs interrupts basement membrane integration, further translating in situ lesion into invasive cancer^21^. Other members of MMP family may involve in extracellular matrix degradation and acquiring invasive ability as well^22^. Besides, hydroxyapatite increases transcription of IL-1β, which subsequently increases MMP-1 and cyclooxygenase (COX)-2 expression (subjected to Northern analysis), with the latter catalyzing prostaglandin (PG) E2 production^20,23^.

Since recent studies have suggested a connection between genes involved in osteoblast differentiation and breast carcinogenesis, we also evaluated CXCR4, MMP-1, CTGF, FGF5, and IL11, all of which were suggested by Kang et al. as being interrogated in breast cancer osteolytic metastasis through microarray profiling and Northern blot confirmation^24^. These candidate genes encode secreted and cell surface proteins. CXCR4 is a bone-homing chemokine receptor, while FGF5 and CTGF are angiogenesis growth factors and IL-11 induces osteoclast from progenitor cells^25–28^. Supplementary Table 1 detailed relevant genes of mammary microcalcification from literature reviews.

All relevant genes were retrieved and the corresponding Affymetrix probe sets were annotated with NetAffx^29^. Boxplot of RMA-normalized expression values of each candidate gene was created between arrays with and without microcalcification with unpaired two-samples Wilcoxon rank sum test. A public domain microarray dataset (GSE2109) with clinical microcalcification status reported was evaluated as an additional validation study.

### Multi-gene signature for microcalcification

Genes with differential mRNA abundance based on the existence of microcalcification were identified, using two-sample T-test with permutation *P* values estimated from 10,000 random permutations to correct for multiple testing from high-dimensional microarray data. Nominal significance level of each univariate test was set to 0.001 to reduce type I error. The same procedure of filtering significant genes was repeated for DCIS as well as comedo-type DCIS. Gene ontology (GO) terms of cellular component, molecular function, and biological process were investigated. Only GO classes and parent classes with at least 5 observations in the selected subset and with an observed versus expected (O/E) ratio of at least 2 were reported. The Gene Set Comparison Tool implemented in the BRB Array Tools was used for pathway analysis while a GO category was evident if the corresponding LS or KS resampling *P* value was below 0.005^30^.

For multi-gene signature construction, filtered genes were used to predict clinical phenotype by multiple methods including compound covariate predictor, diagonal linear discriminative analysis, 3 nearest neighbors, nearest centroid, and support vector machine with default penalty of LIBSVM. Clinical phenotype (histopathology proved microcalcification) was regarded as the gold standard when predictive accuracy was evaluated through leave one out cross-validation. Two-way hierarchical clustering of filtered genes and breast cancer samples was performed with average linkage and Euclidean distance. All bioinformatics works were conducted with the BRB-ArrayTools^30^.

### Breast cancer risk predictive model

A breast cancer risk predictive model was built based on supervised principal component regression^31^. Candidates genes were those identified as being prognostic by univariate Cox regressions. Significant genes with an α level < 0.001 were used for principal components (supergenes) synthesis, with the first principal component incorporated into the breast cancer risk predictive model. Relapse-free survival, with tumor recurrence or metastasis as the first relapse event, was predicted with clinical ER, HER2 status as covariates, and a continuous prognostic index score, which was calculated for each patient by the first principal component score. The high- and low-risk groups were dichotomized by the 50^th^ percentile of estimated prognostic index score.

## Results

### Study population

Breast cancers diagnosed and operated between 2010 and 2014 with curative intention were recruited. A total of 109 breast cancers were successfully assayed with Affymetrix Human Genome U133 Plus 2.0 microarrays. Molecular subtyping with Prediction Analysis of Microarray 50 (PAM50) single sample predictor (SSP) tabulating with IHC results and relapse-free survival between centroid-based luminal-A, luminal-B, basal-like, and HER2-enriched subtype (log-rank test: 0.07) were detailed in Table 1 and Supplementary Fig. 1, respectively^32^.

**Table 1:** Distributions of IHC results and PAM50 molecular subtypes.

IHC results	Single sample predictor			
	Basal-like	HER2-enriched	Luminal-A	Luminal-B
HR+/HER2+	3	5	5	3
HR−/HER2−	4	3	28	18
HR+/HER2+	5	10	1	0
HR−/HER2−	7	3	0	0

Some patients without clinical HER2 status or HER2:2 + but without ISH testing were discarded.

*HR* hormone receptor, *HER2* human epidermal growth receptor type 2.

The presence of microcalcification was documented for each subject. Eighty-six (79%) of the 109 breast cancers had microcalcification identified from histopathological examination. Of these 86 patients, 81 (94%) were with coexistent DCIS. On the other hand, only 13 (57%) of the 23 patients without microscopic microcalcification had their invasive tumors co-grown with DCIS (χ^2^-test, *P* < 0.001, Table 2). High-grade necrotic (comedo type) DCIS were observed in 44 (54%) of the 81 breast cancers with histologically confirmed microcalcifications and coexistent DCIS whereas only 15% (n = 2) of the 13 breast cancers with DCIS but without histological microcalcification, the DCIS lesions were of comedo type (χ^2^-test, *P* < 0.01, Table 3).

**Table 2:** Distributions of DCIS and histopathology-confirmed microcalcification.

Histopathology	Breast cancer		
	Without DCIS	With DCIS	Total
Without microcalcification	10 (43.5%)	13 (56.5%)	23
With microcalcification	5 (5.8%)	81 (94.2%)	86

**Table 3:** Distributions of comedo type DCIS and histopathology-confirmed microcalcification.

Histopathology	Breast cancer with DCIS		
	Non-comedo DCIS	Comedo DCIS	Total
Without microcalcification	11 (84.6%)	2 (15.4%)	13
With microcalcification	37 (45.7%)	44 (54.3%)	81

### mRNA abundance of microcalcification-relevant genes

**Figure 1:**
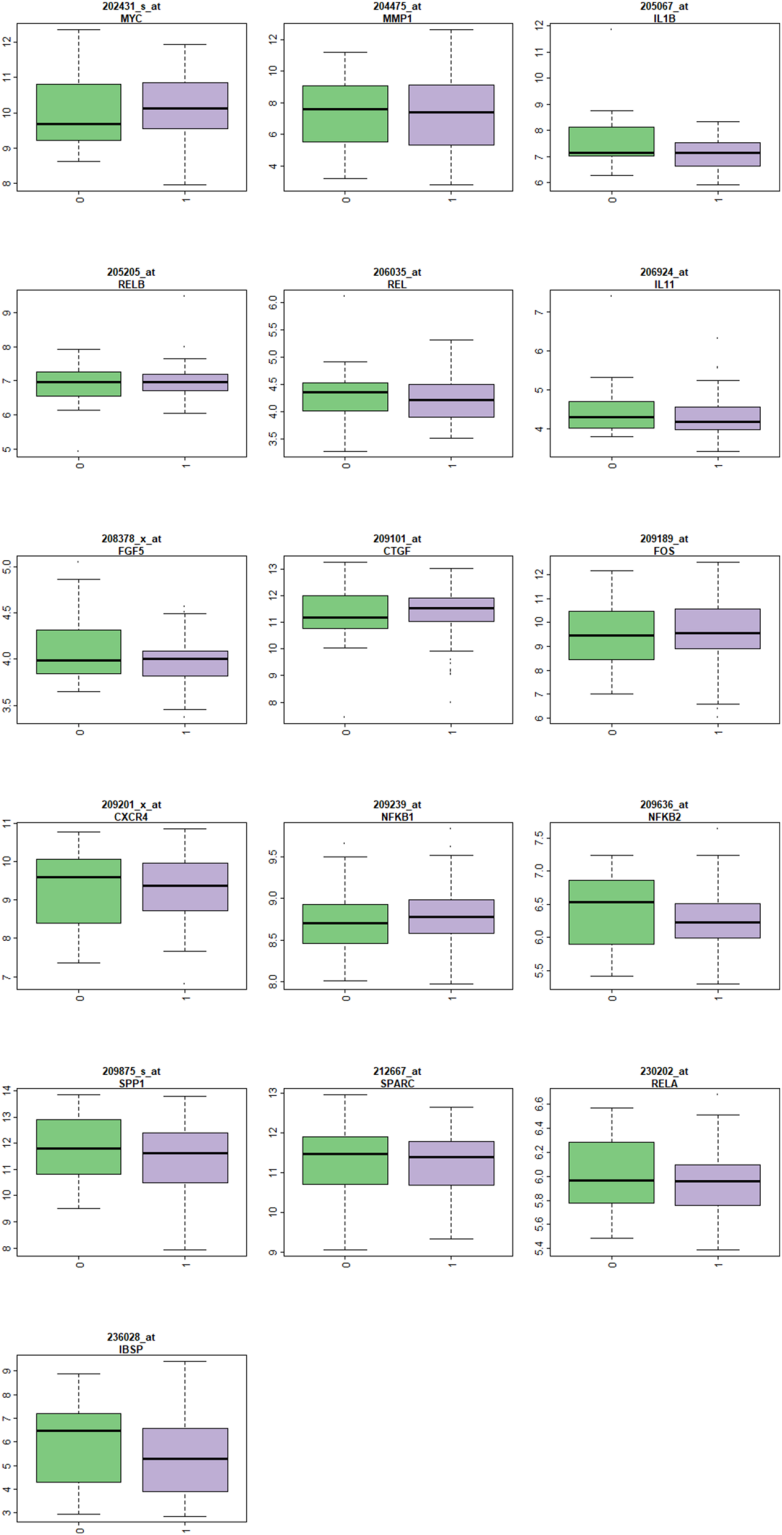
Boxplots of microcalcification-relevant genes from literature reviews. Each plot includes mRNA abundance for one gene stratified by the class variable in X-axis (0: without microcalcification and 1: with microcalcification). The Y-axis represents log intensity and the title shows gene symbol. All comparisons were insignificant with *P* values > 0.001 (Wilcoxon rank sum test).

Figure 1 displayed boxplots of candidate microcalcification-relevant genes from literature reviews. There was no significant transcriptional discrepancy of *SPARC*, *SPP1*, *IBSP*, *NFKB1*, *NFKB2*, *REL*, *RELA*, *RELB*, *FOS*, *MYC*, *IL1B*, *CXCR4*, *MMP-1*, *CTGF*, *FGF5*, and *IL11* between breast cancer patients with and without histology-confirmed microcalcification (unpaired two-samples Wilcoxon rank sum test, all *P* values > 0.001). Supplementary Fig. 5 showed lack of transcriptional difference among these genes from the public domain expO dataset (GSE2109), of which 265 breast cancers with known microcalcification status were assayed.

### Multi-gene signature for breast cancer microcalcification

**Figure 2:**
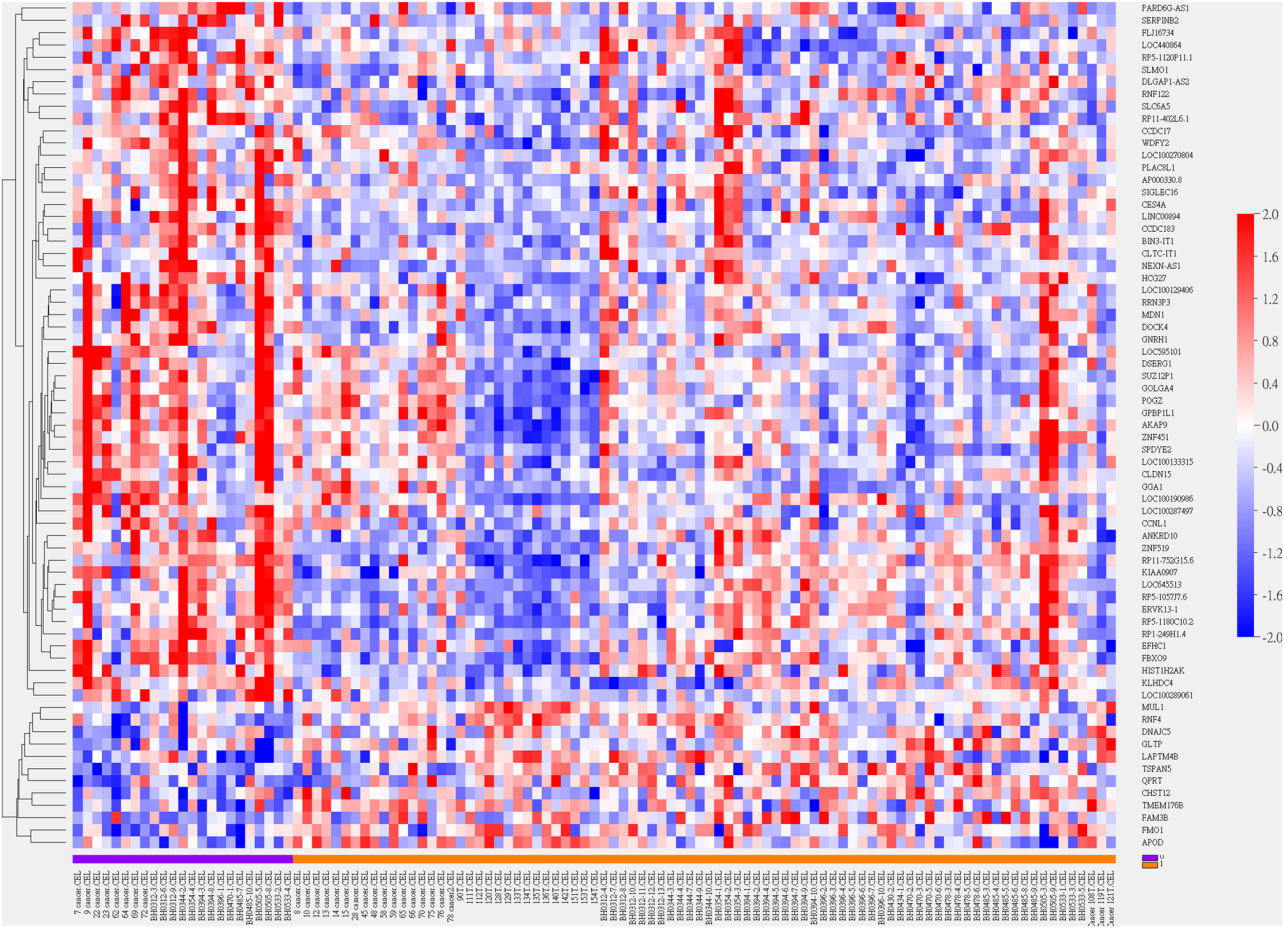
Heatmap of hierarchical clustering of 69 genes with differential mRNA abundance pertaining pathology-confirmed microcalcification. Sample name and class variable (0: without microcalcification (purple) and 1: with microcalcification (brown)) were detailed in X-axis. Gene symbols were listed in Y-axis.

Sixty-nine genes with differential mRNA abundance pertaining pathological diagnosis of microcalcification passed the filter criterion (Fig. 2). Higher mRNA transcription of *GNRH1*, *GGA1*(Lysosome), *CLDN15* and lower mRNA transcription of *QPRT* (Nicotinate/Nicotinamide), *LAPTM4B* (Lysosome), and *DNAJC5* (HSP40) were significantly coincided with the presence of microcalcification (Supplementary Fig. 2). Enriched GO terms were Golgi apparatus (cellular component), glycosaminoglycan, aminoglycan metabolism, and protein ubiquitination (biological process) with O/E ratios of 2.34, 7.85 and 5.64 reported (all resampling *P* values < 0.005), indicating an active secretory process.

**Figure 3:**
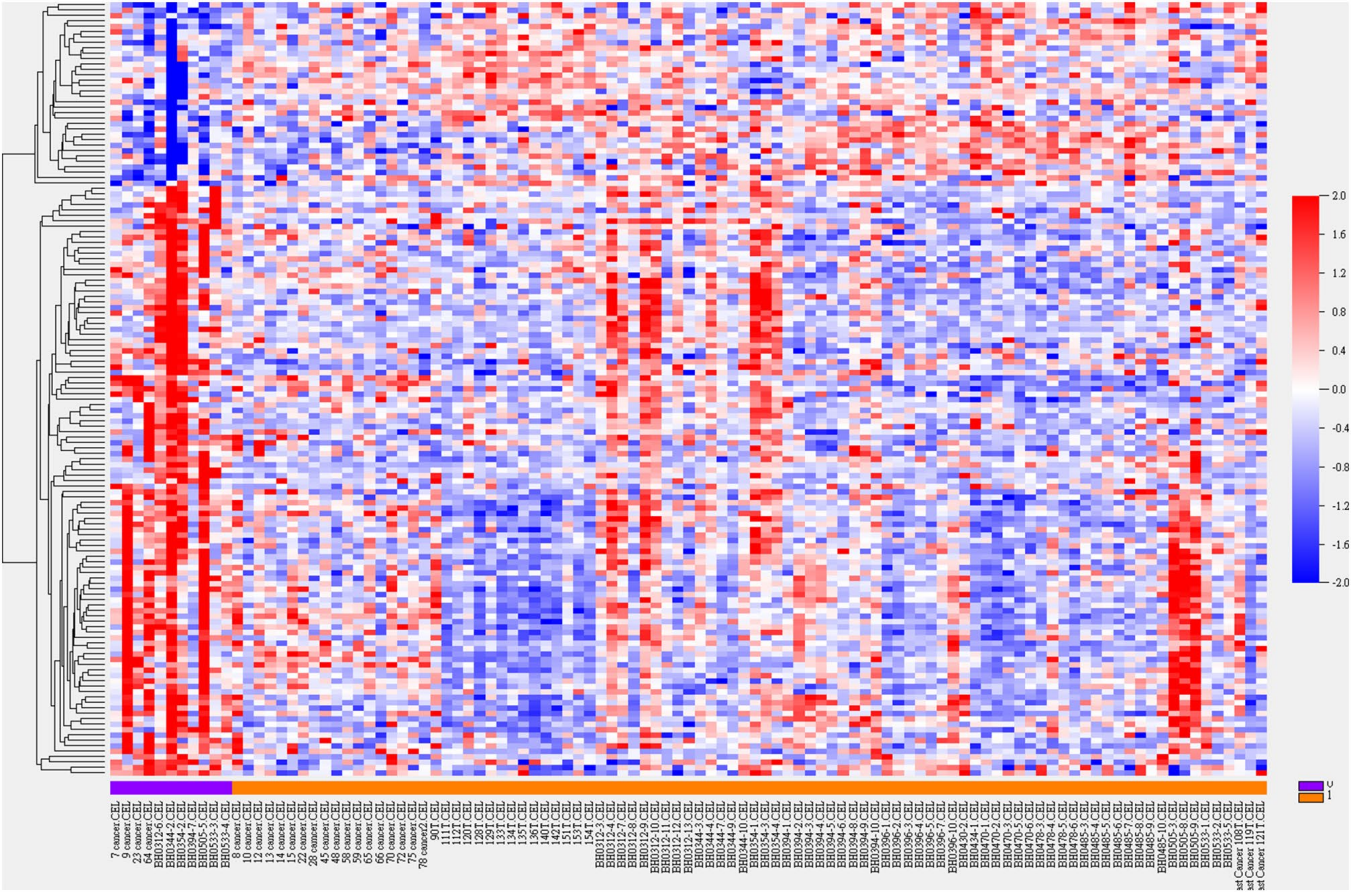
Heatmap of hierarchical clustering of 143 genes with differential mRNA abundance pertaining breast cancer with co-grown DCIS. Sample name and class variable (0: without DCIS (purple) and 1: with DCIS (brown)) were detailed in X-axis. Gene symbols were listed in Supplementary Information File due to limited space.

There were 143 genes with differential mRNA abundance between breast cancer with and without co-growth of DCIS including *XRCC2* (homologous recombination), *GNRH1*, *CACNA1B* (calcium signaling, MAPK pathway), *PRKAA1* (mTOR pathway), *DUSP22* (MAPK pathway), *MKNK1* (MAPK/mTOR pathway), and *SSBP1* (homologous recombination, mitochondria). Figure 3 and Supplementary Fig. 3 depicted clustering heatmap and volcano plot, respectively. Enriched GO pathways with resampling *P* values less than 0.005 were endosome membrane (cellular component, O/E ratio: 2.32), serine-type endo-peptidase activity, carbohydrate binding, calcium ion binding (molecular function, O/E ratios: 6.05, 4.17, 2.16), and Golgi vesicle transport (biological process, O/E ratio: 4.81). If comedo type DCIS was selected as classifying variable, 11 significant genes were identified, including *CCDC183*, *SLMO1*, *SLC6A5*, *CES4A*, *APOD*, *FMO1* (P450 pathway), and *QPRT*.

Due to highly correlated phenotype of DCIS and microcalcification (χ^2^-test, *P* < 0.001), it was intuitive to adopt the intersection of genes with differential mRNA abundance from these two clinical variables. A Bayesian compound covariate predictor classifier was built, and the intersection (18 probe sets) provided the best predictive accuracy with cross-validated Receiver Operating Characteristic (ROC) area under the curve (AUC) achieving 0.713 (Supplementary Fig. 4 and Supplementary Table 2).

### Breast cancer risk predictive model

**Figure 4:**
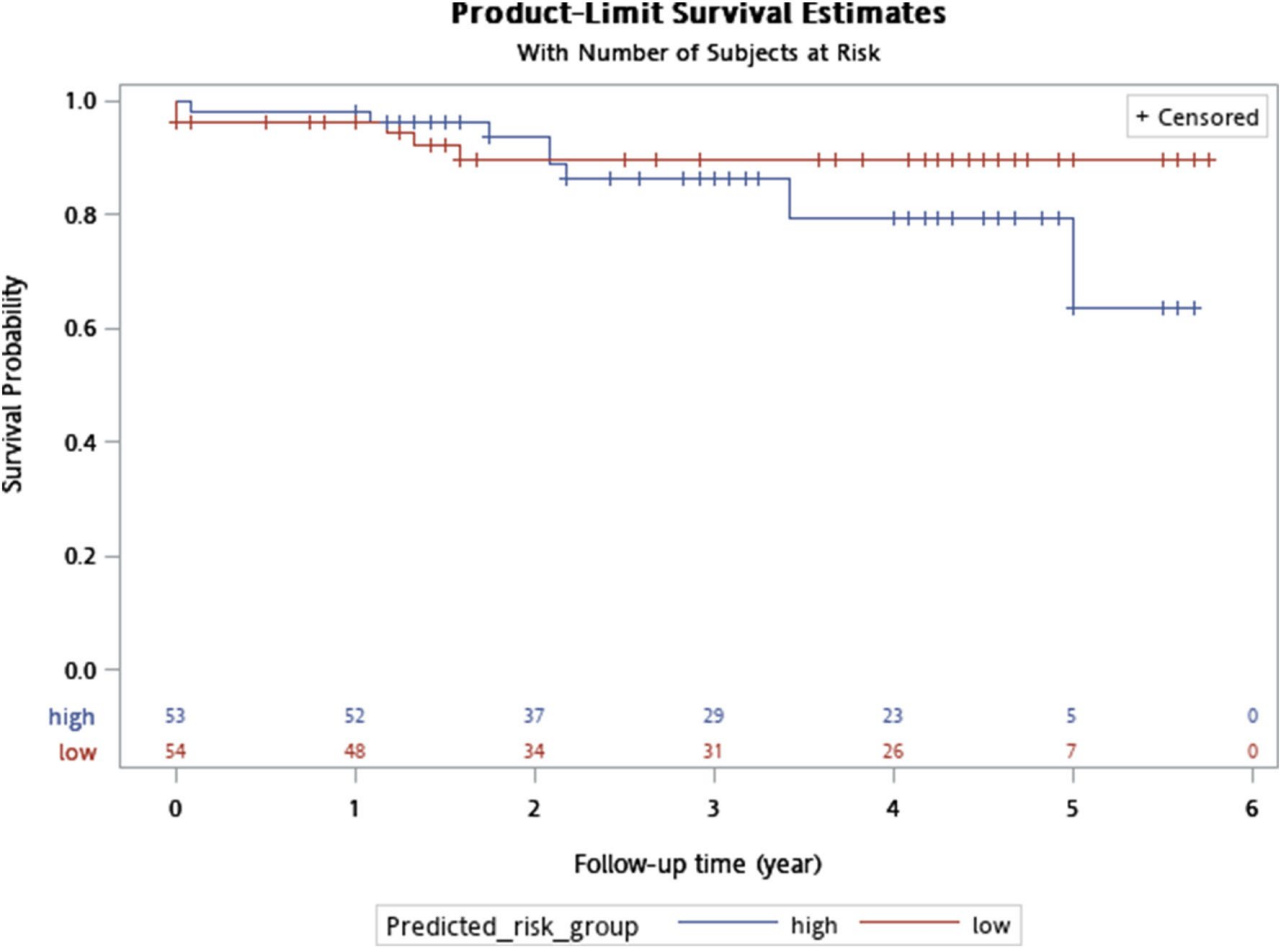
Kaplan–Meier plot for five-year relapse-free survival stratified by high-/low- risk defined by the 50th percentile of prognostic index score calculated from 10 intersection genes pertaining pathological microcalcification and DCIS. X-axis: survival time in year; survival was right-censored at five years.

The intersection of 18 probe sets was further used in relapse-free survival prediction with the 50th percentile of prognostic index score constructing high-/low risk groups. Ten genes were selected by fitting penalized Cox proportional hazards model. The results of leave-one-out cross-validation with clinical ER and HER2 status incorporated as covariates were detailed in Table 4 while Fig. 4 displayed Kaplan–Meier plot. Five-year relapse free survival was 91% for low-risk and 83% for high-risk group (log-rank test, *P* = 0.10). Supplementary Table 3 detailed predicted risk groups and clinical data for survival analysis.

**Table 4:** Genes used in relapse-free survival prediction.

Coefficient	% CV support	Probe set	Symbol
0.1	90.83	1552845_at	CLDN15
1.156	100	1560112_at	WDFY2
− 0.423	99.08	1569320_at	GPBP1L1
− 0.145	97.25	1569484_s_at	MDN1
− 0.792	100	207987_s_at	GNRH1
0.104	95.41	215203_at	GOLGA4
− 0.314	99.08	217671_at	DSERG1
1.231	100	232804_at	AP000330.8
− 0.968	100	239556_at	LOC645513
0.483	100	244840_x_at	DOCK4

*CV* cross-validation.

## Discussion

There remains an unsolved debate on whether mammographic microcalcification is a passive phenomenon resulted from degraded cell debris during mammary gland proliferation or is itself an active process intervening in breast carcinogenesis. For instance, Scimeca et al. hypothesized that microcalcification is the product of mesenchymal-epithelial transformation (EMT), and vimentin, BMP-2, β2-microglobulin, β-catenin, and OPN were proposed as representative IHC markers^10^. In addition to hydroxyapatite, they also found magnesium-substituted hydroxyapatite in vicinity of malignant breast lesions with energy dispersive X-ray microanalysis. Acquiring osteoblast-like phenotype through mesenchymal transformation resulted in microcalcifications.

As early as 1972, Stegner et al. had suggested that mammary calcifications were produced by tumor secretions rather than degenerated cells^33^. As milk is abundant in calcium, it is essential for mammary epithelial cells to concentrate calcium ions^34^. Another possible explanation of ectopic mammary calcifications came from Holme et al. as they argued that aberrant calcium metabolism induced breast microcalcification depositions^35^. It’s not a coincidence that Bellahcène et al. using polyclonal antibodies and immunoperoxidase techniques, identified three bone matrix proteins, namely OPN, OSN, and BSP, which were synthesized by cells with osteoblastic linage and were expressed in human breast cancers, highlighting the importance of bone matrix mineralization in breast carcinogenesis while BSP was postulated to initiate hydroxyapatite formation^16,17^. In addition, aberrant breast BSP expression also correlated with osteotropic bone metastases when measured with immunoperoxidase and specific anti-BSP antibodies from breast cancer cell lines and primary breast cancers metastasizing to bone^16,18^.

Initially we subdivided Taiwanese breast cancer samples based on pathology-proved microcalcification and tested whether there existed any mRNA abundance discrepancy in these relevant genes from literature reviews. These candidate genes were grouped into bone matrix proteins (*SPARC*, *SPP1*, *IBSP*), hydroxyapatite-induced downstream calcium-dependent mitogens (*NFKB1*, *REL*, *RELA*, *RELB, NFKB2*, *FOS*, *MYC*), hydroxyapatide-induced autocrine *IL1B*, and osteoblast differentiation associated genes (*CXCR4*, *MMP-1*, *CTGF*, *FGF5*, and *IL11*)^19,24^. None of these candidate genes showed significant mRNA transcriptional difference between Taiwanese breast cancers with and without histopathogically proved microcalcification. To make sure our experiment results were not incidental findings, we also consulted the expO dataset (GSE2109) while the lack of transcriptional difference further augmenting our negative findings regarding literature-retrieved relevant genes of microcalcification. Therefore, it is necessary to develop a novel multi-gene signature for microcalcification to define the prognostic relevance of ectopic breast mineralization.

Under stringent statistical testing (10,000 permutations with a nominal significance level of 0.001), 69 and 143 genes with mRNA transcriptional discrepancy based on the presence of pathological microcalcification and DCIS were identified. Most of these genes, when inspected individually, did not deliver explicit biological interpretations regarding calcium metabolism or breast precancerous lesion. The multi-gene GO analyses, however, did infer active secretory processes such as Golgi apparatus (cellular component), and biological pathways of glycosaminoglycan, aminoglycan metabolism, and protein ubiquitination pronounced in microcalcification-associated genes. Enriched GO terms for accompanied DCIS differentiated genes included endosome cellular component, serine-type endopeptidase, carbohydrate binding, calcium ion binding functional pathways as well as Golgi vesicle transport process, all of which further reinforced the clinical interconnection and coexistence of microcalcification and DCIS.

It was straightforward to develop a robust and concise multi-gene signature from the intersectional genes pertaining microcalcification and DCIS. We believed transcriptional profiles in terms of mRNA abundance were more sensitive for early detection of pathogenic microcalcification formation and undelaying in situ lesion and a Bayesian compound covariate predictor was proposed with satisfactory performance. Ten genes were further selected to synthesize prognostic index score and relapse-free survival predictive model, which provided independent prognostic power in additional to ER and HER2 status. Although survival discrepancy between high- and low-risk group was of borderline significance (*P* = 0.10), a prognostic trend inherited from microcalcification/DCIS intersection genes was prominent. Constitutional genes with increased breast cancer relapse risk were claudin 15, *WDFY2*, golgin A4, *DOCK4* while *HCG27* and *PLAC8L1* were among 18 signature signs not endorsed by prognostic index score.

There were some limitations of current study. First, the undifferentiated mRNA abundance of literature-suggested microcalcification relevant genes may be biased by Affymetrix probe design. Some candidate genes were reported in IHC assays, immunoperoxidase as well as dispersive X-ray, and transcriptional/translational discrepancy between mRNA and protein measurement inevitably compromised comparability across studies. Second, bone matrix proteins were up-regulated in metastatic breast cancers, especially those with bone metastases. Our samples were of early stage breast cancers without distant metastasis during surgery and might not have these osteotropic genes up-regulated. Third, multi-gene signature for osteolytic bone metastasis from breast cancer cell lines had been published, further studies to recruit more clinical samples including advanced breast cancers with bone metastases should be initiated to elucidate microcalcification deposition and osteotropic metastatic mechanism driven by calcium metabolism^15,36^. Another unanswered argue might arise from whether multi-gene signature can outperform abnormal mammographic readings such as those debrided by BIRADS categories 0, 4, 5 in terms of sensitivity, specificity, and accuracy. Indeed, the oversensitivity and low positive predictive value of mammography resulted in high and unnecessary callbacks and biopsies. A minimally invasive multi-gene signature-based testing from liquid biopsy might be an alternative to current screening modality. Lastly, Current study did not decipher the impact of molecular subtyping (such as IHC4) upon the presence of microcalcification, and future studies with more samples enrolled can evaluate their impact upon mammary calcification^37^.

It deserves notice that the number of cases for microarray experiments was modest only, but details were provided for individual patient (e.g., pathological microcalcification and DCIS status, survival time, relapse indicator, and relevant clinical parameters). Further validation studies for the proposed signature could be conducted by using the most updated method of mRNA quantitation and independent breast cancer patient samples. Nevertheless, our study suggested that mammary microcalcification is not only the earliest detectable radiological sign for breast cancer screening but the phenomenon, ectopic breast mineralization, to some degree, may reflect the underling events during mammary carcinogenesis. Prognostic relevance of the proposed signature might result from relevant biological processes that contribute to the molecular heterogeneity of human breast cancers. Future prospective studies to evaluate the prognostic significance of microcalcification are warranted.

## Supplementary information

Supplementary Information.

## Data Availability

Microarray data in current study was deposited in Gene Expression Omnibus (GEO) with the access number GSE146558.

## References

[1] Elmore JG, Armstrong K, Lehman CD, Fletcher SW (2005). Screening for breast cancer. JAMA.

[2] Yen AM (2016). Population-based breast cancer screening with risk-based and universal mammography screening compared with clinical breast examination: a propensity score analysis of 1429890 Taiwanese women. JAMA Oncol..

[3] U.S. Preventive Services Task Force. Final recommendation statement: breast cancer: screening. *U.S. Preventive Services Task Force* https://www.uspreventiveservicestaskforce.org/Page/Document/RecommendationStatementFinal/breast-cancer-screening1 (2019).

[4] Rao AA, Feneis J, Lalonde C, Ojeda-Fournier H (2016). A pictorial review of changes in the BI-RADS fifth edition. Radiographics.

[5] VanHouten JN (2005). Calcium sensing by the mammary gland. J. Mammary Gland Biol. Neoplasia.

[6] Morgan MP, Cooke MM, McCarthy GM (2005). Microcalcifications associated with breast cancer: an epiphenomenon or biologically significant feature of selected tumors?. J. Mammary Gland Biol. Neoplasia.

[7] Frappart L (1984). Structure and composition of microcalcifications in benign and malignant lesions of the breast: study by light microscopy, transmission and scanning electron microscopy, microprobe analysis, and X-ray diffraction. Hum. Pathol..

[8] Büsing C, Keppler U, Menges V (1981). Differences in microcalcification in breast tumours. Virchows Arch. (Pathol. Anat.).

[9] Spak DA, Plaxco JS, Santiago L, Dryden MJ, Dogan BE (2017). BI-RADS fifth edition: a summary of changes. Diagn. Interv. Imaging.

[10] Scimeca M (2014). Microcalcifications in breast cancer: an active phenomenon mediated by epithelial cells with mesenchymal characteristics. BMC Cancer.

[11] Huang CC (2013). Concurrent gene signatures for han chinese breast cancers. PLoS ONE.

[12] Rosen PP, Hoda SA, Brogi E, Koerner FC (2015). Rosen's Breast Pathology.

[13] Irizarry RA (2003). Exploration, normalization, and summaries of high density oligonucleotide array probe level data. Biostatistics.

[14] Bruford EA (2020). Guidelines for human gene nomenclature. Nat. Genet..

[15] Bellahcène A (2007). Transcriptome analysis reveals an osteoblast-like phenotype for human osteotropic breast cancer cells. Breast Cancer Res. Treat..

[16] Bellahcène A, Merville MP, Castronovo V (1994). Expression of bone sialoprotein, a bone matrix protein, in human breast cancer. Cancer Res..

[17] Bellahcène A, Castronovo V (1995). Increased expression of osteonectin and osteopontin, two bone matrix proteins, in human breast cancer. Am. J. Pathol..

[18] Bellahcène A, Kroll M, Liebens F, Castronovo V (1996). Bone sialoprotein expression in primary human breast cancer is associated with bone metastases development. J. Bone Miner. Res..

[19] McCarthy GM (1998). Molecular mechanism of basic calcium phosphate crystal-induced activation of human fibroblasts. Role of nuclear factor kappab, activator protein 1, and protein kinase c. J. Biol. Chem..

[20] Morgan MP, Cooke MM, Christopherson PA, Westfall PR, McCarthy GM (2001). Calcium hydroxyapatite promotes mitogenesis and matrix metalloproteinase expression in human breast cancer cell lines. Mol. Carcinog..

[21] Nelson AR, Fingleton B, Rothenberg ML, Matrisian LM (2000). Matrix metalloproteinases: biologic activity and clinical implications. J. Clin. Oncol..

[22] Lochter A (1997). Matrix metalloproteinase stromelysin-1 triggers a cascade of molecular alterations that leads to stable epithelial-to-mesenchymal conversion and a premalignant phenotype in mammary epithelial cells. J. Cell Biol..

[23] Rutter JL, Benbow U, Coon CI, Brinckerhoff CE (1997). Cell-type specific regulation of human interstitial collagenase-1 gene expression by interleukin-1 beta (IL-1 beta) in human fibroblasts and BC-8701 breast cancer cells. J. Cell. Biochem..

[24] Kang Y (2003). A multigenic program mediating breast cancer metastasis to bone. Cancer Cell.

[25] Taichman RS (2002). Use of the stromal cell-derived factor-1/CXCR4 pathway in prostate cancer metastasis to bone. Cancer Res..

[26] Giordano FJ (1996). Intracoronary gene transfer of fibroblast growth factor-5 increases blood flow and contractile function in an ischemic region of the heart. Nat. Med..

[27] Moussad EE, Brigstock DR (2000). Connective tissue growth factor: what's in a name?. Mol. Genet. Metab..

[28] Manolagas SC (1995). Role of cytokines in bone resorption. Bone.

[29] Thermo Fisher Scientific. NetAffx Analysis Center. *Thermo Fisher Scientific*. https://www.affymetrix.com/analysis/index.affx (2017).

[30] Simon R (2007). Analysis of gene expression data using BRB-ArrayTools. Cancer Inform..

[31] Bair E, Tibshirani R (2004). Semi-supervised methods to predict patient survival from gene expression data. PLOS Biol..

[32] Parker JS (2009). Supervised risk predictor of breast cancer based on intrinsic subtypes. J. Clin. Oncol..

[33] Stegner H, Pape C (1972). Beitrag zur feinstruktur der sogenanntan mikrokalzifikation in mammatumoren. Zentralbl. Allg. Path..

[34] Ahmed A (1975). Calcification in human breast carcinomas: ultrastructural observations. J. Pathol..

[35] Holme T (1993). Is mammographic microcalcifiication of biological significance?. Eur. J. Surg. Oncol..

[36] Yibin K (2003). A multigenic program mediating breast cancer metastasis to bone. Cancer Cell.

[37] Cuzick J (2011). Prognostic value of a combined estrogen receptor, progesterone receptor, Ki-67, and human epidermal growth factor receptor 2 immunohistochemical score and comparison with the Genomic Health recurrence score in early breast cancer. J. Clin. Oncol..

