# Supplementary material for: Multi-gene signature of microcalcification and risk prediction among Taiwanese breast cancer
Source: Sci Rep. 2020 Oct 26;10:18276. doi: 10.1038/s41598-020-74982-1 (PMC7588423; doi:10.1038/s41598-020-74982-1)
Supplement: Supplementary file 1 — Supplementary Information. [file 41598_2020_74982_MOESM1_ESM.pdf]

Multi-gene Signature of Microcalcification and Risk Prediction among Taiwanese Breast Cancer

Hsin-Tien Tsai, Ching-Shui Huang, Chao-Chiang Tu, Chih-Yi Liu, Chi-Jung Huang, Yuan-Soon Ho, Shih-Hsin Tu, Ling-Ming Tseng, Chi-Cheng Huang

Multi-gene Signature of Microcalcification and Risk Prediction among Taiwanese Breast Cancer

Supplementary information

Supplementary Figure legends

Supplementary Fig 1 Relapse-free survival of Taiwanese breast cancers stratified by PAM50 molecular subtypes. Only subjects with long-term follow up were surveyed. SSP2: single sample predictor without normal breast-like subtype; Basal: basal-like; HER2: HER2-enriched; Lum-A: luminal A; Lum-B: luminal B molecular subtype; X-axis: survival time in year.

Supplementary Fig 2 Volcano plot of Taiwanese breast cancer transcriptome. Blue dots indicated genes with differential mRNA abundance pertaining pathological microcalcification (p-value <0.001).

Supplementary Fig 3 Volcano plot of Taiwanese breast cancer transcriptome. Blue dots indicated genes with differential mRNA abundance pertaining coexistent DCIS (p-value <0.001).

Supplementary Fig 4 Cross-validated Receiver Operating Characteristic (ROC) curve of Bayesian compound covariate predictor (BCCP) classifier for multi-gene signature pertaining microcalcification.

Supplementary Fig 5 Boxplots of microcalcification-relevant genes in independent dataset (GSE2109). Each plot includes mRNA abundance for one gene stratified by the class variable in X-axis (0: without microcalcification and 1: with microcalcification). The Y-axis represents log intensity and the title shows gene symbol. All comparisons were insignificant with p-values >0.001 (Wilcoxon rank sum test).

Gene symbol list (from top to bottom) for Y-axis of Fig 3

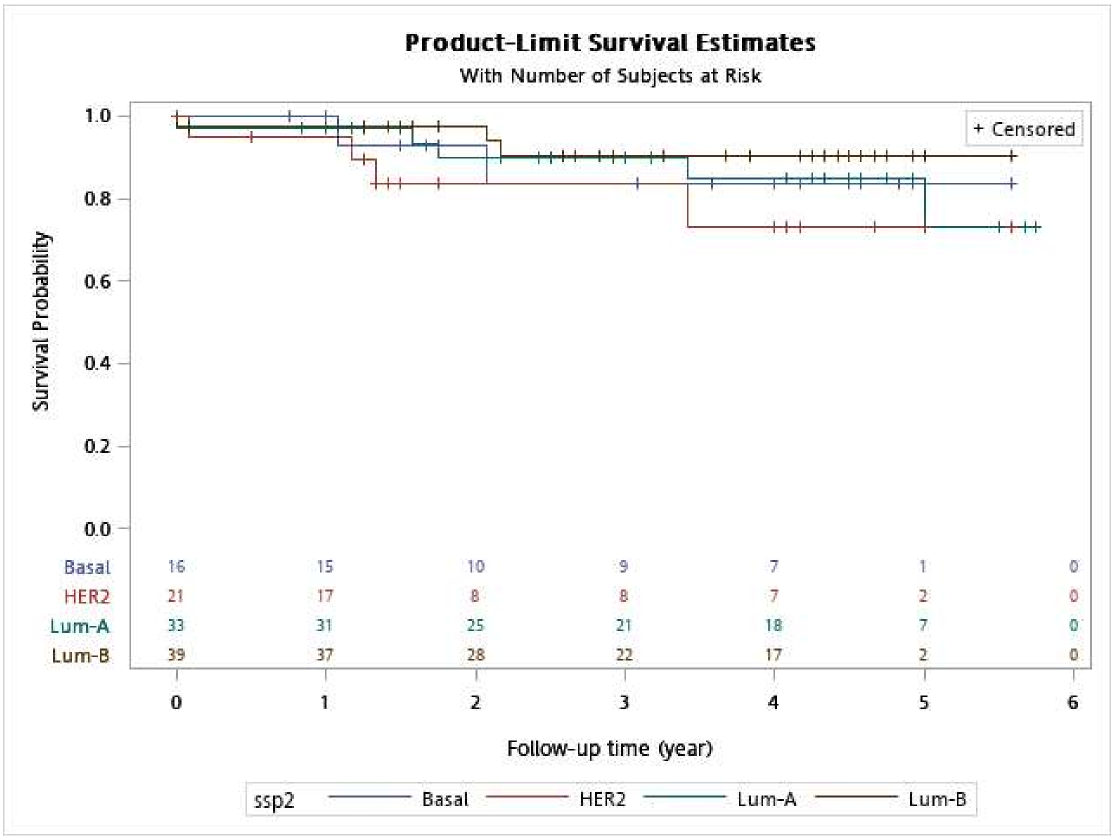

Supplementary Fig 1

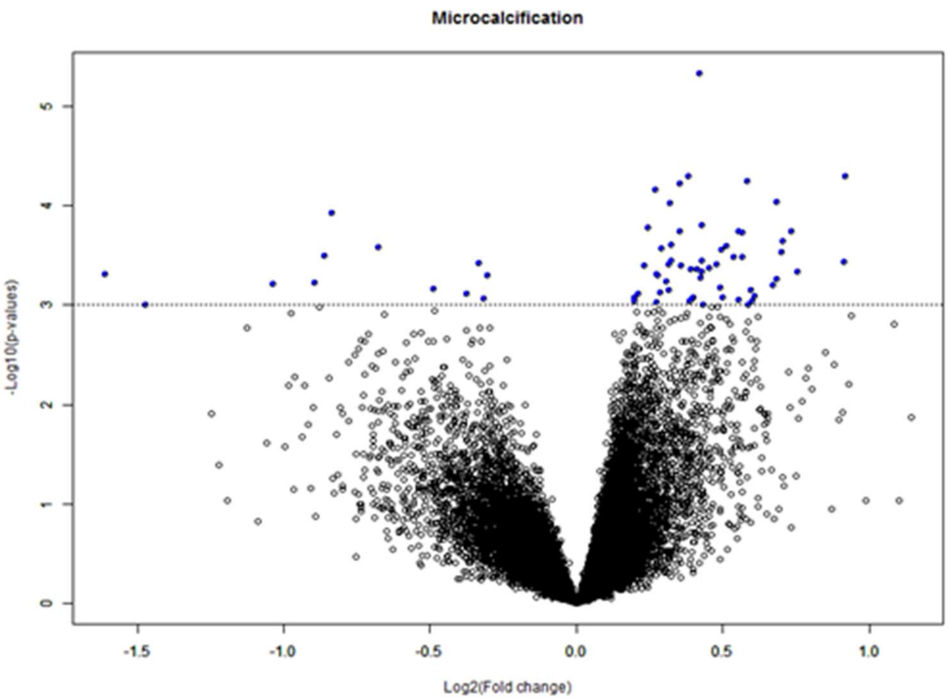

Supplementary Fig 2

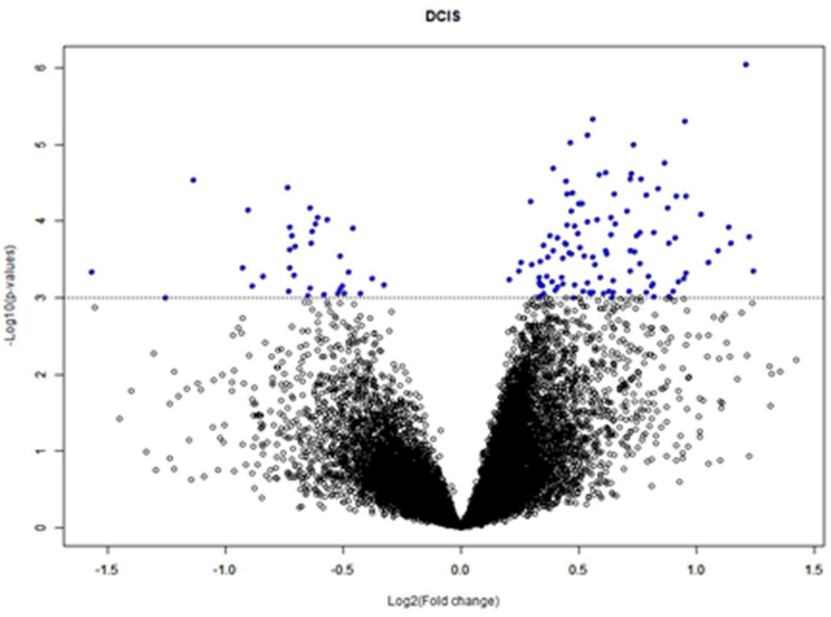

Supplementary Fig 3

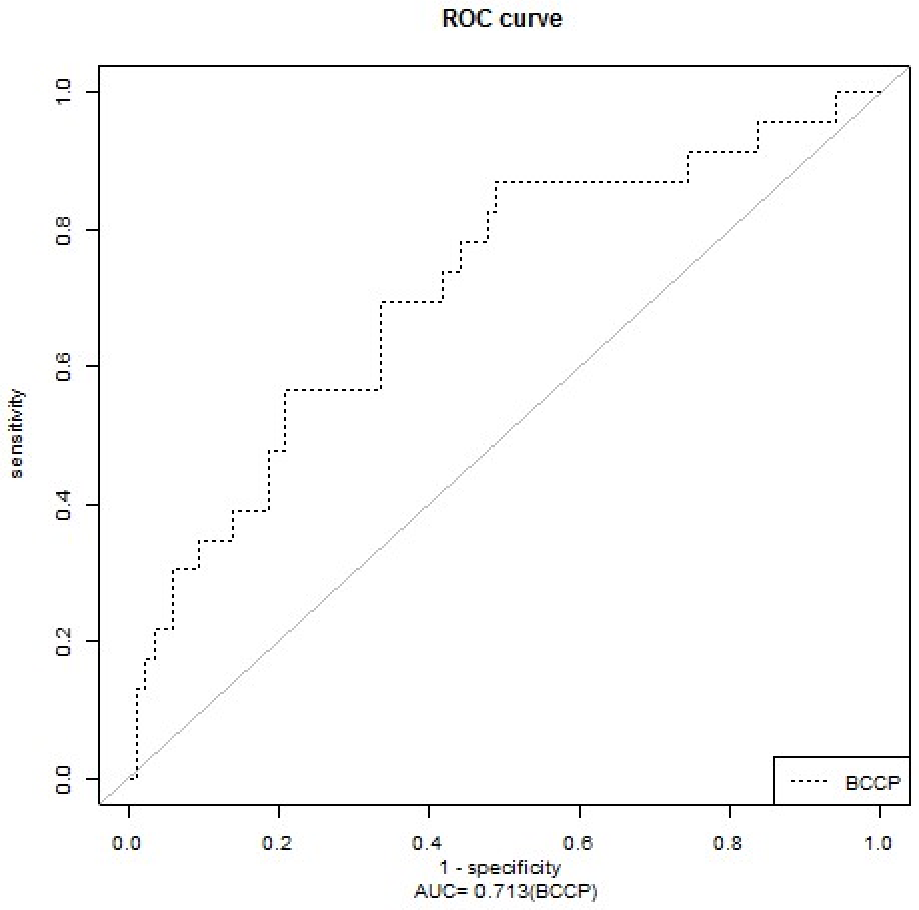

Supplementary Fig 4

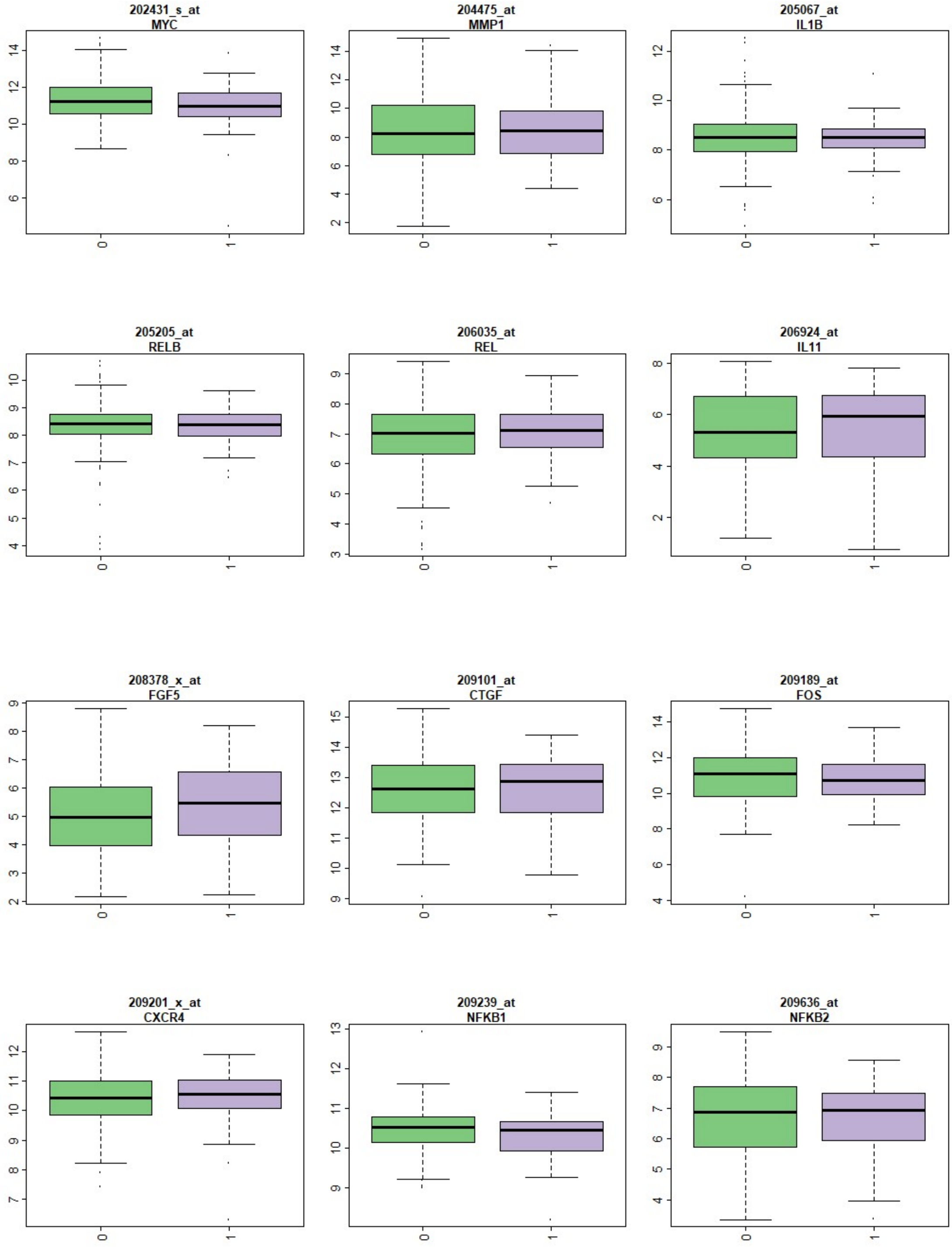
202431_s_at
MYC
204475_at
MMP1
205067_at
IL1B
205205_at
RELB
206035_at
REL
206924_at
IL11
208378_x_at
FGF5
209101_at
CTGF
209189_at
FOS
209201_x_at
CXCR4
209239_at
NFKB1
209636_at
NFKB2
0
1

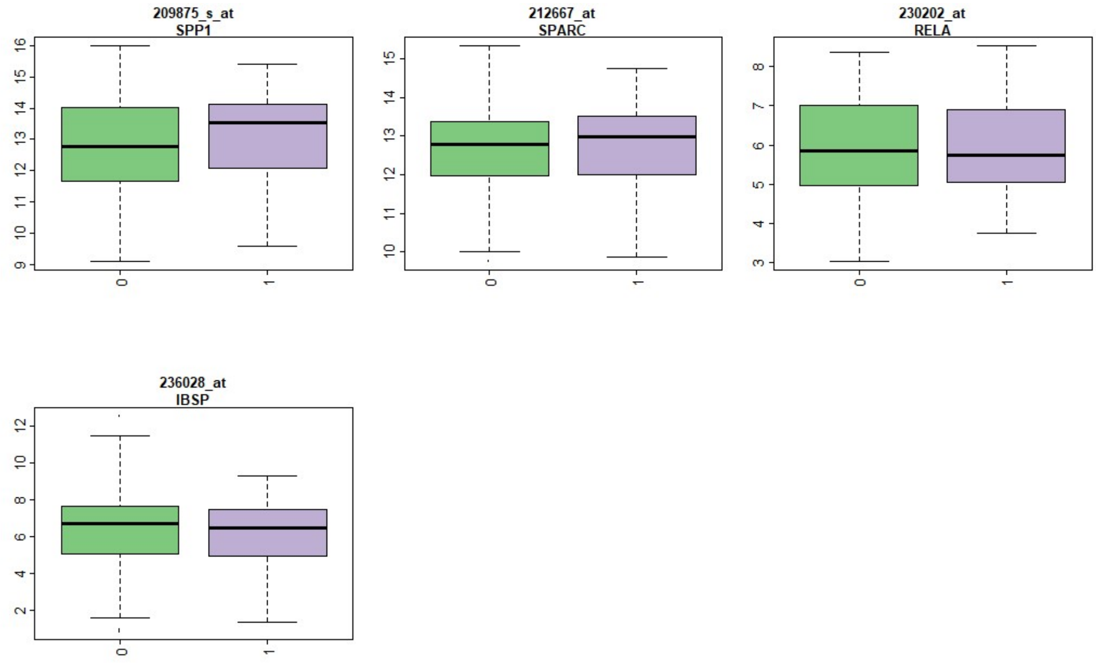

Supplementary Fig 5

Supplementary Table 1. Candidates of microcalcification-relevant genes from literature reviews.

Probe set	Description	Symbol
202431_s_at	v-myc avian myelocytomatosis viral oncogene homolog	MYC [1]
205067_at	interleukin 1, beta	IL1B [2]
205205_at	v-rel avian reticuloendotheliosis viral oncogene homolog B	RELB [1]
206036_s_at	v-rel avian reticuloendotheliosis viral oncogene homolog	REL [1]
207535_s_at	nuclear factor of kappa light polypeptide gene enhancer in B-cells 2 (p49/p100)	NFKB2 [1]
209189_at	FBJ murine osteosarcoma viral oncogene homolog	FOS [1]
209239_at	nuclear factor of kappa light polypeptide gene enhancer in B-cells 1	NFKB1 [1]
209875_s_at	secreted phosphoprotein 1	SPP1 [3,4]
209878_s_at	v-rel avian reticuloendotheliosis viral oncogene homolog A	RELA [1]
212667_at	secreted protein, acidic, cysteine-rich (osteonectin)	SPARC [4]
236028_at	integrin-binding sialoprotein	IBSP [4-7]
208378_x_at	fibroblast growth factor 5	FGF5 [8]
209101_at	connective tissue growth factor	CTGF [8]
209201_x_at	chemokine (C-X-C motif) receptor 4	CXCR4 [8]
206924_at	interleukin 11	IL11 [8]
204475_at	matrix metallopeptidase 1	MMP1 [2,9]

References to Supplementary Table 1

1. McCarthy GM, Augustine JA, Baldwin AS, Christopherson PA, Cheung HS, Westfall PR, Scheinman RI (1998) Molecular mechanism of basic calcium phosphate crystal-induced activation of human fibroblasts. Role of nuclear factor kappab, activator protein 1, and protein kinase c. J Biol Chem. 273:35161-9.
2. Rutter JL, Benbow U, Coon CI, Brinckerhoff CE (1997) Cell-type specific regulation of human interstitial collagenase-1 gene expression by interleukin-1 beta (IL-1 beta) in human fibroblasts and BC-8701 breast cancer cells. J Cell Biochem 66:322-36.
3. Scimeca M, Giannini E, Antonacci C, Pistolese CA, Spagnoli LG, Bonanno E (2014) Microcalcifications in breast cancer: an active phenomenon mediated by epithelial cells with mesenchymal characteristics. BMC Cancer 14:286.
4. Bellahcène A, Bachelier R, Detry C, Lidereau R, Cl?zardin P, Castronovo V (2007) Transcriptome analysis reveals an osteoblast-like phenotype for human osteotropic breast cancer cells. Breast Cancer Res Treat 101:135-48.
5. Bellahcène A, Merville MP, Castronovo V (1994) Expression of bone sialoprotein, a bone matrix protein, in human breast cancer. Cancer Res 54:2823-6.
6. Bellahcène A, Castronovo V (1995) Increased expression of osteonectin and osteopontin, two bone matrix proteins, in human breast cancer. Am J Pathol 146:95-100.Bellahc?ne A, Kroll M, Liebens

F, Castronovo V (1996) Bone sialoprotein expression in primary human breast cancer is associated with bone metastases development. J Bone Miner Res 11:665-70.

7. Bellahcène A, Kroll M, Liebens F, Castronovo V (1996) Bone sialoprotein expression in primary human breast cancer is associated with bone metastases development. J Bone Miner Res 11:665-70.
8. Kang Y, Siegel PM, Shu W, Drobnjak M, Kakonen SM, Cord?n-Cardo C, Guise TA, Massaguè J (2003) A multigenic program mediating breast cancer metastasis to bone. Cancer Cell 3:537-49.
9. Morgan MP, Cooke MM, Christopherson PA, Westfall PR, McCarthy GM (2001) Calcium hydroxyapatite promotes mitogenesis and matrix metalloproteinase expression in human breast cancer cell lines. Mol Carcinog 32:111-7.

Supplementary Table 2. Intersect of differentially expressed genes pertaining microcalcification and DCIS.

**Probe set**	**Description**	**Symbol**
1552845_at	claudin 15	CLDN15
1559050_at	HLA complex group 27 (non-protein coding)	HCG27
1560112_at	WD repeat and FYVE domain containing 2	WDFY2
1569315_s_at	long intergenic non-protein coding RNA 894	LINC00894
1569320_at	GC-rich promoter binding protein 1-like 1	GPBP1L1
1569484_s_at	MDN1, midasin homolog (yeast)	MDN1
207287_at	BIN3 intronic transcript 1 (non-protein coding)	BIN3-IT1
207987_s_at	gonadotropin-releasing hormone 1 (luteinizing-releasing hormone)	GNRH1
215203_at	golgin A4	GOLGA4
217671_at	Down syndrome encephalopathy related protein 1	DSERG1
218246_at	mitochondrial E3 ubiquitin protein ligase 1	MUL1
232804_at	uncharacterized LOC100506385	AP000330.8
236320_at	coiled-coil domain containing 17	CCDC17
237783_at	PLAC8-like 1	PLAC8L1
238412_at	RNA polymerase I transcription factor homolog (S. cerevisiae) pseudogene 3	RRN3P3
239556_at	uncharacterized LOC645513	LOC645513
240868_at	uncharacterized LOC100129406	LOC100129406
244840_x_at	dedicator of cytokinesis 4	DOCK4

Supplementary Table 3. Predicted risk groups and clinical data for survival analysis of 109 Taiwanese breast cancers

Microarray experiment names	DCIS	Comed-DCIS	Microcalcification	Survival time(year)	Relapse status	ER	PR	Grade	HER2 IHC(ISH)	HER2 status	Predicted risk group
7 cancer.CEL	0		0	5.58	0	80	10	3	2	0	low
8 cancer.CEL	1	1	1	1.58	1	0	0	3	3	1	low
9 cancer.CEL	0		0	5.75	0	85	50	2	1	0	low
10 cancer.CEL	1	0	1	5.50	0	80	50	1	1	0	high
12 cancer.CEL	1	0	1	5.67	0	90	0	1	2	0	low
13 cancer.CEL	1	1	1	2.08	1	0	0	3	2	0	high
14 cancer.CEL	1	1	1	5.58	0	0	0	2	3	1	low
15 cancer.CEL	1	1	1	5.50	0	70	20	3	3	1	low
22 cancer.CEL	1	0	0	5.58	0	0	0	3	1	0	high
23 cancer.CEL	0		0	5.67	0	80	90	2	2	0	low
28 camcer.CEL	1	0	1	5.67	0	70	30	2	2	0	high
45 cancer.CEL	1	0	1	5.00	1	0	0	2	1	0	high
48 cancer.CEL	1	1	1	4.00	0	0	0	3	3	1	high
58 cancer.CEL	1	1	1	4.75	0	70	15	3	3	1	low
59 cancer.CEL	1	1	1	3.42	1	50	40	3	1	0	high
62 cancer.CEL	0		0	1.33	1	0	0	3	3	1	low
64 cancer.CEL	0		0	1.75	1	0	0	3	2	0	high
65 cancer.CEL	1	1	1	5.00	0	80	0	3	3	1	low

66 cancer.CEL	1	1	1	4.92	0	90	50	2	3	1	low
69 cancer.CEL	0		0	4.92	0	0	0	3	1	0	high
70 cancer.CEL	1	0	1	4.92	0	95	95	2	2	0	low
72 cancer.CEL	1	1	0	5.00	0	0	0	3	3	1	high
75 cancer.CEL	1	1	1	4.67	0	0	0	3	3	1	high
76 cancer.CEL	0		1	4.75	0	80	60	2	1	0	low
78 cancer2.CEL	1	1	1	4.83	0	0	0	3	1	0	high
90T.CEL	1	1	1	4.50	0	10	10	3	1	0	high
111T.CEL	1	1	1	4.17	0	95	15	2	1	0	high
112T.CEL	1	0	1	4.08	0	95	95	1	2	0	high
120T.CEL	1	1	1	0.08	1	0	0	3	2	0	high
128T.CEL	1	0	1	3.67	0	95	95	3	3	1	low
129T.CEL	1	0	1	3.67	0	95	60	2	3	1	low
133T.CEL	1	1	1	3.58	0	0	0	3	3	1	low
134T.CEL	1	1	1	3.08	0	0	0	3	3	1	high
135T.CEL	1	0	1	2.08	1	70	10	3	1	0	high
136T.CEL	1	0	1	3.17	0	95	95	2	1	0	high
140T.CEL	1	1	1	2.17	0	0	0	2	3	1	high
142T.CEL	1	1	1	1.25	0	0	0	3	3	1	low
151T.CEL	1	1	1	4.17	0	60	30	2	3	1	low
153T.CEL	1	1	1	1.75	0	0	0	2	3	1	high
154T.CEL	1	1	1	2.67	0	80	90	2	3	1	low
BH0312-3.CEL	1	0	0	4.58	0	95	60	2	3	1	low

BH0312-4.CEL	1	0	1	4.58	0	20	10	2	2	0	high
BH0312-6.CEL	0		0	4.58	0	0	0	3	3	1	low
BH0312-7.CEL	1	1	1	1.17	1	0	0	3	3	1	low
BH0312-8.CEL	1	1	1	4.25	0	75	75	3	1	0	high
BH0312-9.CEL	1	0	0	4.25	0	90	90	3	1	0	high
BH0312-10.CEL	1	0	1	4.17	0	80	80	2	2	0	high
BH0312-11.CEL	1	1	1	4.08	0	95	95	3	2	0	high
BH0312-12.CEL	1	0	1	3.83	0	40	10	3	3	1	low
BH0312-13.CEL	1	1	1	1.08	1	0	0	3	2	0	high
BH0344-2.CEL	0		0	4.50	0	95	40	2	1	0	high
BH0344-3.CEL	1	0	1	4.50	0	95	30	2	3	1	low
BH0344-4.CEL	1	0	1	3.42	1	15	0	2	2	0	high
BH0344-7.CEL	1	0	1	3.83	0	95	70	2	3	1	low
BH0344-9.CEL	1	0	1	2.83	0	95	95	2	2(1.02)	0	high
BH0344-10.CEL	1	0	1	2.58	0	95	0	2	2(0.82)	0	high
BH0354-1.CEL	1	0	1	4.58	0	90	0	3	2	0	low
BH0354-2.CEL	0		1	4.17	0	0	0	2	3	1	low
BH0354-3.CEL	1	1	1	2.17	1	95	15	3	1	0	high
BH0354-4.CEL	1	0	0	2.92	0	10	0	3	2(1.14)	0	high
BH0394-1.CEL	1	1	1	4.67	0	90	50	2	2	0	low
BH0394-2.CEL	1	1	1	4.17	0	0	0	2	3	1	low
BH0394-3.CEL	1	0	0	4.50	0	95	25	1	2	0	low
BH0394-4.CEL	1	0	1	4.50	0	95	10	2	1	0	low

BH0394-5.CEL	1	1	1	4.42	0	95	30	2	2	0	low
BH0394-6.CEL	1	0	1	4.33	0	95	10	3	2	0	low
BH0394-7.CEL	0		1	4.25	0	95	5	3	3	1	low
BH0394-8.CEL	1	0	0	3.00	0	95	30	2	2(1.1)	0	high
BH0394-9.CEL	1	0	1	2.92	0	90	40	1	1	0	low
BH0394-10.CEL	1	0	1	0.50	0	0	0	2	3	1	low
BH0396-1.CEL	1	0	0	4.67	0	50	60	2	0	0	high
BH0396-2.CEL	1	1	1	4.08	0	60	5	3	3	1	low
BH0396-3.CEL	1	0	1	4.25	0	95	50	2	2	0	low
BH0396-4.CEL	1	1	1	0.08	0	95	1	3	3	1	low
BH0396-5.CEL	1	1	1	3.25	0	80	5	3	2	0	high
BH0396-6.CEL	0		1	4.33	0	80	60	2	1	0	high
BH0396-7.CEL	1	0	1	2.42	0	95	30	2	0	0	high
BH0396-10.CEL	1	1	1	2.50	0	70	30	2	3	1	low
BH0430-2.CEL	1	1	1	0.50	0	0	0	3	3	1	low
BH0434-1.CEL	1	0	1	0.00	1	90	20	2	2(1.76)	0	low
BH0470-1.CEL	1	0	0	1.75	0	90	50	2	1	0	high
BH0470-2.CEL	1	1	1	1.75	0	0	0	3	3	1	high
BH0470-3.CEL	1	0	1	1.75	0	9	9	3	1	0	high
BH0470-5.CEL	1	0	1	1.50	0	90	30	2	3	1	low
BH0470-6.CEL	1	1	1	1.42	0	70	70	3	1	0	low
BH0478-3.CEL	1	0	1	1.50	0	90	90	2	1	0	low
BH0478-4.CEL	1	0	1	1.50	0	90	30	2	1	0	low

BH0478-5.CEL	1	0	1	1.25	0	90	90	2	2(1.06)	0	high
BH0478-6.CEL	1	1	1	1.17	0	0	0	3	1	0	high
BH0485-3.CEL	1	0	1	1.50	0	90	0	2	2(3.32)	1	low
BH0485-4.CEL	1	1	1	1.42	0	0	0	3	3	1	high
BH0485-5.CEL	1	0	1	1.42	0	90	10	2	1	0	low
BH0485-6.CEL	1	1	1	1.42	0	1	0	3	3	1	high
BH0485-7.CEL	1	0	0	1.42	0	90	3	1	1	0	low
BH0485-8.CEL	1	0	1	0.00	1	95	98	3	1	0	low
BH0485-9.CEL	1	1	1	1.25	0	0	0	3	3	1	high
BH0485-10.CEL	1	0	0	1.33	0	1	0	2	3	1	high
BH0505-3.CEL	1	0	1	1.00	0	95	60	2	1	0	low
BH0505-5.CEL	0		0	1.00	0	0	0	3	2(1.95)	0	high
BH0505-8.CEL	1	1	0	0.00	0	0	0	3	3	1	low
BH0505-9.CEL	1	1	1	1.67	0	20	0	3	1	0	low
BH0533-1.CEL	1	1	1	0.75	0	98	0	3	1	0	low
BH0533-2.CEL	1	0	0	1.58	0	90	60	2	2(2.21)	1	low
BH0533-3.CEL	0		1	1.50	0	1	0	2	3	1	high
BH0533-4.CEL	0		0	1.58	0	90	5	2	1	0	high
BH0533-5.CEL	1	1	1	0.83	0	60	5	3	3	1	low
Breast Cancer 108T.CEL	1	1	1	4.33	0	0	0	2	3	1	high
Breast Cancer 119T.CEL	1	0	1	4.25	0	95	40	2	2	0	high

Breast Cancer 121T.CEL	1	0	1	4.00	0	0	0	2	3	1	high

.

Gene symbol list (from top to bottom) for Y-axis of Fig 3

Symbol

EHD3

SLC22A17

IGFBP2

C19orf25

STK16

ZDHHC16

HPS6

ZBTB9

NOP9

MUL1

HTRA2

GLTPD1

SCAMP2

FDFT1

RAI1

DNAL4

YWHAQ

TOR1B

FAM213B

KRT71

TMEM203

ATRN

DCP1B

ALG2

ENOPH1

KIAA1279

SLC25A38

CINP

TMEM25

DNAJC16

OGFOD3

TMEM242

S100A14

PRDX2

SLC17A6

TLR8-AS1

TKTL1

IL1RAP

CPA4

NFE4

AGO3

DUSP22

CCDC17

CTRL

LOC284009

PSORS1C3

AP000330.8

GAS6-AS1

PLAC8L1

GUCY1B2

PPIEL

RP1-17K7.2

RP3-365O12.2

LINC00113

DKFZp667F0711

PRR26

CNTNAP3

LINC00639

LINC00973

LINC00485

LGSN

FLJ37786

ALX1

ZNF876P

NECAB2

KRT85

GK5

LINC00328

OR10D3

RNF213

FAHD2CP

CYP1A2

HIST1H1T

KCNK9

EMR3

GPR97

LINC00302

IL20RB

SERPINB7

SLCO4A1

NWD2

ANGPTL4

PKD1L2

EIF1

LSMEM1

PPFIA4

MAGEA4

CACNA1B

LOC101060391

RHBDL1

RRN3P2

HCG27

XRCC2

WDFY2

GPBP1L1

MARK3

GRB10

MYL6

GNRH1

BIN3-IT1

LINC00893

LINC00894

NOMO3

MDN1

ARHGEF10

RRN3P3

MKNK1

KIAA0754

NRD1

UGP2

FAM185A

PRO2852

GOLGA4

CFLAR

DSERG1

LOC100129406

TMEM43

KATNBL1

LOC100507311

LOC100131541

PCSK7

TMEM63A

ECHDC2

EIF3B

SSBP1

ARF1

CLDN15

LINC00152

RALGAPA2

FAM13A-AS1

DOCK4

PRKAA1

LOC645513

LINC01125

UQCRC2

LOC100507281

CREB5

ASAP1-IT1

FLJ21369

MAEL

NQO2

SCML1

SCML2
